# Dataset of mouse hippocampus profiled by LC–MS/MS for label-free quantitation

**DOI:** 10.1016/j.dib.2015.12.057

**Published:** 2016-02-15

**Authors:** Jane A. English, Caitriona Scaife, Akiko Harauma, Melanie Focking, Kieran Wynne, Gerard Cagney, Toru Moriguchi, David R. Cotter

**Affiliations:** a Department of Psychiatry, Royal College of Surgeons in Ireland, ERC Beaumont Hospital, Dublin, Ireland; b Proteome Research Centre, School of Medicine and Medical Science, UCD Conway Institute of Biomolecular and Biomedical Research, University College Dublin, Ireland; c Department of Food and Life Science, Azabu University, Sagamihara, Japan

**Keywords:** Mouse, Brain, Hippocampus, Proteins, Proteomics, Mass spectrometry, Omega-3 deficiency

## Abstract

This dataset reports on the analysis of mouse hippocampus by LC–MS/MS, from mice fed a diet that was either deficient in n-3 FA (n-3 Def) or sufficient in n-3 FA (n-3 Adq). Label free quantitative (LFQ) analysis of the mass spectrometry data identified 1008 quantifiable proteins, 115 of which were found to be differentially expressed between the two dietary groups (*n*=8 per group). This data article refers to the research article “Omega-3 fatty acid deficiency disrupts endocytosis, neuritogenesis, and mitochondrial protein pathways in the mouse hippocampus” (English et al., 2013 [1]), in which a more comprehensive interpretation and analysis of the data is given.

**Specifications table**

**Table:** 

Subject area	*Biology*
More specific subject area	*Proteomics, neurobiology*
Type of data	*Raw mass spectrometry files (.raw), processed data files (txt.zip)*
How data was acquired	*LC–MS/MS was performed on an LTQ Orbitrap XL mass spectrometer (Thermo Fisher Scientific) coupled to an Ultimate 3000 RSLC Ultra High Pressure Liquid Chromatography system (Thermo Scientific Dionex).*
Data format	*Raw MS/MS files (PRIDE archive), supplementary descriptive tables (Excel)*
Experimental factors	*Omega-3 Fatty acid deficient diet*
Experimental features	*Mouse hippocampus tissue was sub-pooled according to gender (4 male and 4 female per dietary group). Following solubilisation 50 μg sample was denatured, alkylated and subjected to overnight digestion by trypsin. Digested peptides were run in triplicate on an LTQ Orbitrap XL mass spectrometer. Label-free quantitation on the mass spectrometer data files was performed with MaxQuant.*
Data source location	*NA*
Data accessibility	*Mass spectrometry data files (.RAW), MaxQuant LFQ output (.txt) and Perseus statistical analysis (.txt) have been uploaded to ProteomeXchange Consortium via the PRIDE partner repository with the dataset identifier **PXD001277.** The MaxQuant and Perseus files are located in the zipped Search file txt.zip*

## **Value of the data**

• Comprehensive list proteins that map to the hippocampus sub-region of mouse brain
• Comprehensive list of proteotypic peptides for each protein identified from the mouse hippocampus, to enable future targeted proteomic workflows in this sub-region.
• These data could be used for comparison with other sub-regions or sub-proteomes of mouse brain tissue
• Provides a valuable reference for future characterisation studies of brain proteins that are sensitive to changes in dietary omega-3 fatty acids.

## 1 Data, experimental design, materials and methods

### 1.1 Experimental design

For label free LC–MS/MS analysis, the hippocampus of 16×n-3 Def and 16×n-3 Adq samples per dietary group were assessed. Prior to analysis, these 16 samples were sub-pooled according to gender (4 males and 4 females per dietary group) so that 8×n-3 Def and 8×n-3 Adq LC–MS/MS runs were performed.

### 1.2 Sample preparation

Following solubilisation of samples by sonication in triethylammonium bicarbonate (TEAB; Sigma), 50 µg of protein from each of the 8×n-3 Adq and 8×n-3 Def samples was denatured for 10 min at 80 °C in the presence of 1% RapiGest (Waters). Samples were reduced with TCEP (50 mM) for 60 min at 60 °C, followed by alkylation with Iodoacetamide (200 mM) for 30 min in the dark. Digestion with trypsin (Promega; 1 µg/µl) was carried out overnight at 37 °C, and Tryptic peptides were purified using ZipTips (Millipore) according to the manufacturer׳s instructions.

### 1.3 LC–mass spectrometry

Samples were re-suspended in Solution A (HPLC grade water, 2% acetonitrile, 0.5% acetic acid) whereby 1 µg of protein digest (5 µl) was injected per LC–MS/MS run. Each of the 16 samples was run in triplicate, on an LTQ ORBITRAP XL (Thermo Fisher Scientific) mass spectrometer connected to an Ultimate 3000 RSLCnano liquid chromatography system (Thermo Scientific Dionex). Each sample was loaded onto Biobasic Picotip Emitter (120 mm length, 75 μm ID) packed in house with Reprocil Pur C18 (1.9 μm) reverse phase media column, and was separated by an increasing acetonitrile gradient, using a 56 min reverse phase gradient at a flow rate of 250 nL/min. From 0 to 7 min 5 μl (1 µg) of sample was injected and loaded onto the column at a flow rate of 1.5 μl/min. At 7 min the mass spectrometer started to acquire data, the chromatography gradient continued, 7–44 min from 5% to 35% Solution B (acetonitrile containing 3% HPLC grade water and 0.5% acetic acid) at 250 nl/min, 44–50 min from 35% to 90% Solution B at 250 nl/min, 50–55 min at 90% Solution B at 250 nl/min increasing to 900 nl/min, 55–56 min at 90% Solution B to 2% Solution B at 900 nl/min decreasing to 250 nl/min. The mass spectrometer was operated in positive ion mode with a capillary temperature of 200 °C, a capillary voltage of 45 V, a tube lens voltage of 100 V and with a potential of 1800 V applied to the frit. Survey full-scan MS spectra (300–2000 Da) were acquired in the Orbitrap with a resolution of 60,000, whereby the FTMS maximum injection time was 700 ms, the Ion trap injection time was 100 ms, and the 7 most intense ions from the preview scan were selected for MS/MS analysis.

### 1.4 Data processing

Protein identification and label-free quantification (LFQ) was performed with MaxQuant (V 1.1.1.36) searching against the IPI mouse.v3.68 fasta formatted database (release December 2009). Methionine oxidations and acetylation of protein N-termini were specified as variable modifications, while carbamidomethylation was specified as a fixed modification, and 2 missed cleavages were allowed. Protein and peptide FDR׳s were set to 0.01. Only proteins with at least two peptides (one uniquely assignable to the protein) were considered as reliably identified. LFQ intensity values were used for protein quantification between groups. Only unique and razor peptides were considered for quantification with a minimum ratio count of 2. Statistical analyses was performed in Perseus (V 1.3.0.4), whereby the data was log 2 transformed, missing values were replaced by values form the normal distribution, and column normalisation was performed by subtracting the median. Student׳s *t*-test was applied to identify proteins differentially expressed between the two dietary groups at a 5% threshold, and a permutation-based FDR was applied at a 5% threshold.

## Role of funding source

David R. Cotter is funded by the Health Research Board, Clinical Scientist Award. Ger Cagney is funded by Science Foundation Ireland.

## Appendix A Supplementary material

Supplementary material

Supplementary material Supplemental Table 1—Dietary Group assignment

Supplementary material Supplemental Table 2—Summary table (Quantified protein list, LFQ *p*-value, fold change)
