# Supplementary material for: Dataset of mouse hippocampus profiled by LC–MS/MS for label-free quantitation
Source: Data Brief. 2016 Feb 15;7:341–3. doi: 10.1016/j.dib.2015.12.057 (PMC4781930; doi:10.1016/j.dib.2015.12.057)
Supplement: Supplementary file 1 — Supplementary material [file mmc1.docx]

The authors declare no conflict of interest
